# Purebred and Crossbred Genomic Evaluation and Mate Allocation Strategies To Exploit Dominance in Pig Crossbreeding Schemes

**DOI:** 10.1534/g3.120.401376

**Published:** 2020-06-18

**Authors:** David González-Diéguez, Llibertat Tusell, Alban Bouquet, Andres Legarra, Zulma G. Vitezica

**Affiliations:** * GenPhySE, Université de Toulouse, INRAE, ENVT, F-31326, Castanet Tolosan, France,; ^†^ IFIP Institut du Porc, BP35104, 35651 Le Rheu, France, and; ^‡^ France Génétique Porc, BP35104, 35651 Le Rheu, France

**Keywords:** Purebred and crossbred evaluation, non-additive genetic effects, heterosis, purebred-crossbred genetic correlation, Genomic Prediction, GenPred, Shared Data Resources

## Abstract

We investigated the effectiveness of mate allocation strategies accounting for non-additive genetic effects to improve crossbred performance in a two-way crossbreeding scheme. We did this by computer simulation of 10 generations of evaluation and selection. QTL effects were simulated as correlated across purebreds and crossbreds, and (positive) heterosis was simulated as directional dominance. The purebred-crossbred correlation was 0.30 or 0.68 depending on the genetic variance component used. Dominance and additive marker effects were estimated simultaneously for purebreds and crossbreds by multiple trait genomic BLUP. Four scenarios that differ in the sources of information (only purebred data, or purebred and crossbred data) and mate allocation strategies (mating at random, minimizing expected future inbreeding, or maximizing the expected total genetic value of crossbred animals) were evaluated under different cases of genetic variance components. Selecting purebred animals for purebred performance yielded a response of 0.2 genetic standard deviations of the trait “crossbred performance” per generation, whereas selecting purebred animals for crossbred performance doubled the genetic response. Mate allocation strategy to maximize the expected total genetic value of crossbred descendants resulted in a slight increase (0.8%, 4% and 0.5% depending on the genetic variance components) of the crossbred performance. Purebred populations increased homozygosity, but the heterozygosity of the crossbreds remained constant. When purebred-crossbred genetic correlation is low, selecting purebred animals for crossbred performance using crossbred information is a more efficient strategy to exploit heterosis and increase performance at the crossbred commercial level, whereas mate allocation did not improve crossbred performance.

Crossbreeding schemes are widely used in almost all species of livestock production, especially in monogastric species (pigs and birds in particular). The main goal of crossbreeding is to improve the performance of crossbred (CB) animals by exploiting heterosis and breed complementarity (Falconer 1981). Dominance is one of the major genetic bases of heterosis and mate allocation can be used to maximize the total genetic merit of future progeny by exploiting dominance variation across-breeds (*e.g.*, crosses in a 2-way crossbreeding scheme) and within-breed (*e.g.*, in a purebred population) (DeStefano and Hoeschele 1992; Hayes and Miller 2000; Toro and Varona 2010). In pigs, although the selection is made within purebreds (PB) (Dekkers 2007), the commercial CB animals can be created by selecting specific pairs of mates between breeds that result in a superior CB descendants in terms of performance compared to random mating. With the advent of high-density single nucleotide polymorphism (SNP), genomic selection has become a standard practice in the genetic evaluation of livestock populations (Meuwissen *et al.* 2013). Moreover, nowadays, estimating dominance effects in genetic evaluations has become feasible in a genomic BLUP (best linear unbiased prediction) context (Vitezica *et al.* 2013). Thus, SNP-based mate allocation strategies accounting for non-additive genetic effects have been developed (Toro and Varona 2010). Such strategies have provided encouraging results to maximize the expected total genetic merit of future progeny within breed, on computer simulation (Toro and Varona 2010) and on real data in dairy cattle and pigs (Ertl *et al.* 2014; Aliloo *et al.* 2017; González-Diéguez *et al.* 2019). However, the benefits of genomic mate allocation strategies to increase performance in a crossbreeding scheme have not been evaluated in the long term in a genomic scheme.

In crossbreeding schemes, the main limitation to improve the performance of CB animals by selection on PB is that the genetic correlation ($r_{PC}$) between PB and CB performances is lower than 1 (*e.g.*, 0.63 on average in pigs, with 50% of the estimates between 0.45 and 0.87 (Wientjes and Calus 2017)). This low genetic correlation between PB and CB may be due to genotype-by-environment interaction (GxE), and genotype-by-genotype interactions (GxG) (*i.e.*, dominance and/or epistasis). The effects of genetic causal variants depend on the environment where the animal is raised (GxE), and depend on the genetic backgrounds where the variants are expressed in (GxG). Both, GxE and GxG may result in a low $r_{PC}$ between PB and CB (Wientjes and Calus 2017; Duenk *et al.* 2020). If $r_{PC}$ is low, genetic merit of PB parents evaluated in a PB population are a poor predictor of the performance of their CB descendants (Dekkers 2007). Then, the integration of both PB and CB information is essential in genetic evaluation oriented to improve CB performance (Wei and van der Werf 1994). Although several genomic models have been proposed to address these issues (Dekkers 2007; Zeng *et al.* 2013; Esfandyari *et al.* 2015a), only Xiang *et al.* (2016) addressed most of the issues influencing the level of $r_{PC}$, in particular the inclusion of additive and dominance effects, and use of PB and CB data simultaneously. Their model fits purebred and crossbred data together fitting additive and dominance effects in a multivariate manner (Varona *et al.* 2010; Karoui *et al.* 2012). Xiang *et al.*’s model also includes a regression on “genomic inbreeding” (observed homozygosity) in PB and CB animals to measure individual “inbreeding depression”. This can be seen as the opposite of heterosis and therefore it is possible to correct by, and to predict, individual heterosis (Iversen *et al.* 2019). In this manner, all essential aspects of the joint PB – CB prediction are considered in a single analysis.

Although the methodology is available for simultaneously accounting for all these aspects in genomic evaluations there is a need of addressing the benefits of its implementation in practice in a crossbreeding scheme. This is crucial because its implementation would require large organizational changes in the way pig breeding schemes are organized today.

The objective of this study was to investigate the effectiveness of mate allocation strategies and genomic evaluations that account for additive and dominance effects to improve CB performance. Breed-specific QTL and individual genomic heterosis effects were explicitly simulated in a two-way pig crossbreeding scheme. The effects of the sources of information used in the genetic evaluation (only PB data or PB and CB data), of several narrow and broad-sense heritability values, and of several options for mate allocation to produce the CB were examined.

## Materials And Methods

To evaluate the effectiveness of mate allocation strategies on crossbreeding, a two-way pig crossbreeding selection scheme for a maternal trait (*e.g.*, litter size) was simulated. The simulation was split into two steps. In the first step, the simulator QMSim (Sargolzaei and Schenkel 2009) was used to generate a historical population structure. In the second step, a self-made Fortran program was developed to simulate a two-way maternal pig crossbreeding scheme across ten generations, and evaluate four different scenarios. The scenarios differed in the sources of information used to evaluate the selection candidates (PB or PB and CB) and in the use or not of mate allocation strategies to produce the CB descendants. Different values of genetic (co)variance components were also tested.

### Historical and recent populations

**Figure 1:**
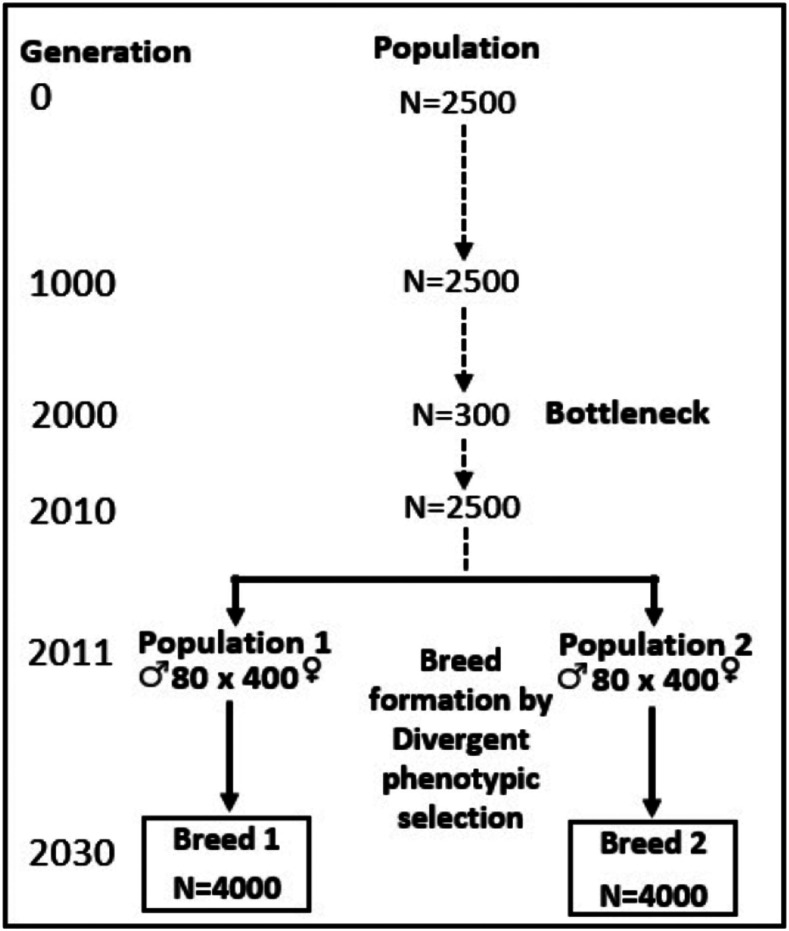
Schematic representation of the simulated historical population (First step). N stands for number of animals.

To create the historical population (HP) (undergoing drift and mutation), the simulator QMSim (Sargolzaei and Schenkel 2009) was used. Figure 1 shows a schematic representation of the simulated historical population. First, a constant population size of 2500 individuals was generated over 1000 generations of random mating. Second, from generation 1001 to 2000, the population size was gradually reduced to 300 individuals in order to simulate a bottleneck and generate initial linkage disequilibrium (LD). Then, 10 additional generations were simulated to gradually expand the size of the population from 300 to 2500 individuals. At the historical generation (2010), there were equal numbers of males and females (1250 each sex). Two samples of 480 animals (80 males and 400 females) were drawn from the generation 2010 to create two breeds, P1 and P2. Then, the two breeds were divergently selected based on phenotype for 20 generations. Within each breed, selected animals were mated with a restriction to reduce inbreeding during breed formation. A litter size of 10 was used. In the last generation of breed formation (generation 2030), 4000 animals (with an equal number of males and females) composed each breed. Note that a dummy phenotype with a narrow-sense heritability of 0.5 was used in the first step, only in order to create the linkage disequilibrium structure in parental breeds.

**Figure 2:**
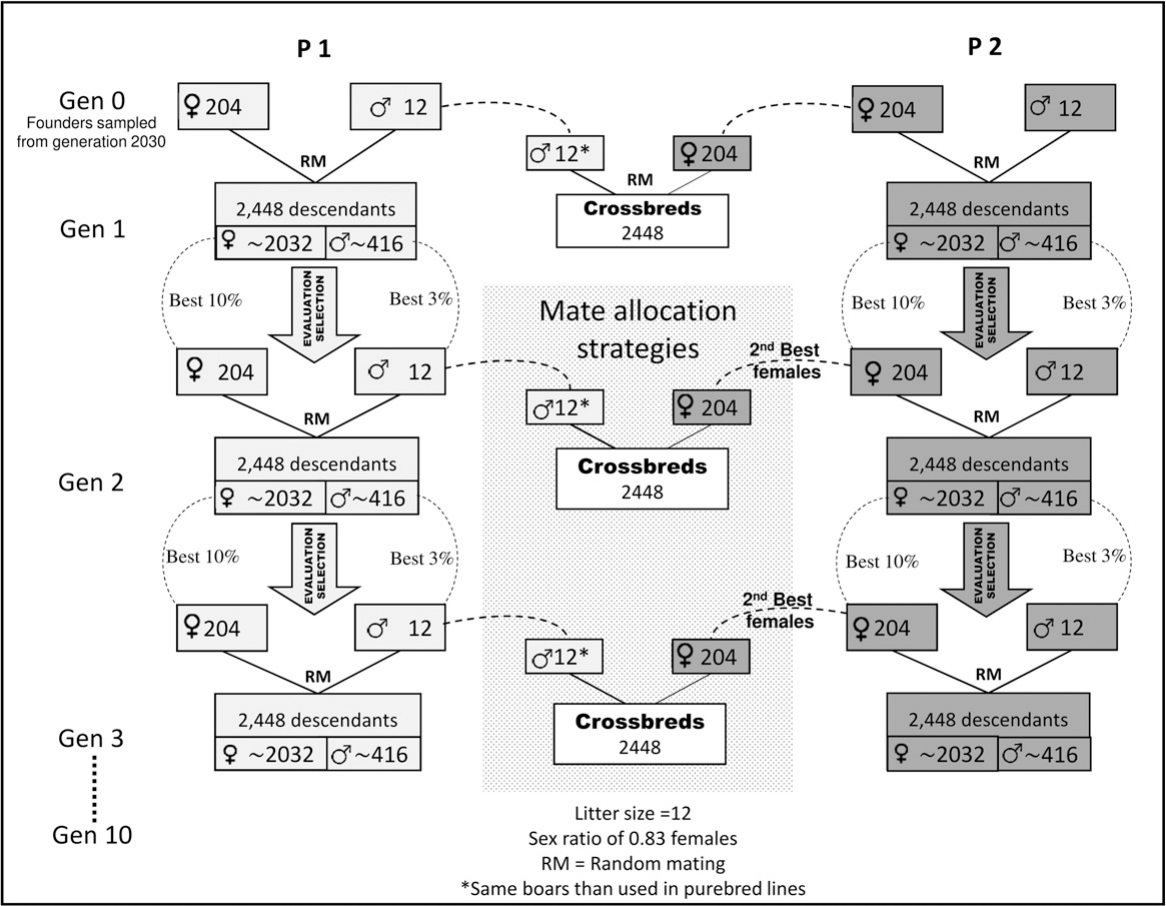
Schematic representation of the simulated two-way crossbreeding scheme (second step). Genetic evaluations and selection were carried out in each generation within breed. Best purebred animals were selected to be mated at random to produce the next generations within breed. The same best males selected to create the next generations within P1 were also crossed with the second-best females from P2 to create the crossbred descendants. Mate allocation strategies were implemented only to create the crossbred animals.

**Figure 3:**
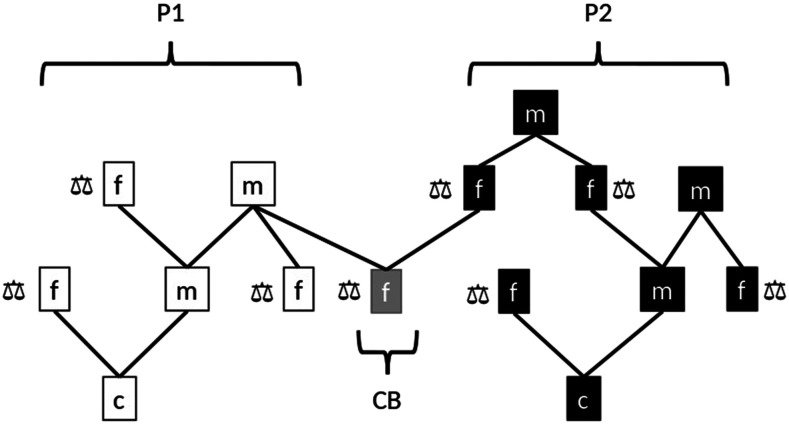
Close sources of information available through pedigree to evaluate the candidates to selection (c) via males (m) and females (f) in the two breeds (P1 and P2). A “balance” symbol means recorded phenotype. CB stands for crossbreds.

In the second step, a two-way pig crossbreeding scheme with 10 generations of selection was simulated (Figure 2). The initial generations of breeds P1 and P2 were formed by randomly sampling 12 males and 204 females (founders) from each respective breeds in generation 2030. First generation of P1 (and P2) was mated at random to produce the first progeny of PB animals. From generation 1 to 10, PB animals were evaluated and selected based on different models and criteria depending on each scenario (see description below). Within each breed, selected animals were mated at random to produce the next generation. Real pigs breeding schemes are complex with several steps of selection, and its simulation is not straightforward. To simplify programming, we used a litter size of 12 with an artificial sex ratio of 0.83 females, resulting in 2448 descendants in each generation (∼2032 females and ∼416 males, Figure 2). Selection intensity was 3% and 10% with respect to the simulated number of males and females, respectively, which could correspond to those used in a pig breeding scheme after performing pre-selection of individuals on other traits (*e.g.*, morphological defects, disease resistance, etc). Hence, the best 12 males and 204 females were chosen within each breed at each generation to be parents of the next generation of PB animals. These animals were selected at birth (before having any own record) based on selection criteria that will be detailed later. Figure 3 shows the closest sources of information available to evaluate the candidates to selection of the two parental breeds.

To create the CB population, P1 was used as sire breed and P2 was used as dam breed. The first CB progeny was created by crossing at random the 12 founder males from P1 with the 204 founder females from P2. These CB animals are half-sibs of PB animals in generation 1. In subsequent generations, CB progenies were created mating the 12 best males selected within P1 with the 204 second best candidate females from P2. Note that since the best 204 best females were already used within P2, they were not available for generating CB animals. Then, different mate allocation strategies to generate the CB progeny were implemented depending on each scenario (see below). All PB and CB females were eventually phenotyped and genotyped, but at the time of selection, candidates to selection were not phenotyped. The information available for the estimation of breeding values at the time of selection differed between breeds. For a selection candidate in P1, the most related CB animal with a performance record was an offspring of the paternal grandsire (*i.e.*, with an additive genetic relationship of 0.125). For a selection candidate in P2, the most related CB animal with a performance record was an offspring of a paternal grand-grand-sire (*i.e.*, with an additive genetic relationship of 0.03125). This difference occurs because in P2, different sets of dams were selected to produce PB and CB offspring, while in P1, the same set of sires was used to create both PB and CB offspring (Figure 3). Hence, the breeding scheme was not symmetric. All the simulation steps (historical and recent populations) were replicated 10 times.

### Genome

The genome consisted of 18 chromosomes (autosomes) of 120 cM each. In the first generation of the historical population, biallelic markers (72,000) and QTL (7,200) were distributed at random along the chromosomes with 0.5 allele frequencies. The mutation rate (recurrent mutation process) for markers and QTL was assumed to be $2.5\times 10^{-4}$ per locus per generation.

Marker quality control was done in each breed (generation 2030) independently. Markers with minor allele frequency (MAF) lower than 0.05 and out of Hardy-Weinberg equilibrium (t > 0.15, based on Wiggans *et al.* (2009)) were unqualified. Only markers that passed quality control in both breeds were kept. In that way, the number and order of markers were similar in both breeds. In founder generation (generation 0) of P1 and P2 breeds, around 50K SNPs (in each replicate) were segregating in both breeds.

Linkage disequilibrium patterns (LD) were also evaluated in the two breeds (P1 and P2) in generation 2030. LD was measured by calculating the squared correlation coefficient ($r^{2}$) between all pairs of markers (Hill 1974). The LD decay was measured for increasing distances between markers by calculating the mean $r^{2}$ within each distance interval. The resulting average over replicates of LD of SNP with an interval distance, assuming a 1Mbp:1cM ratio, of 0.9 to 1 Mbp, was the same for both parental breeds, $r^{2}=$ 0.15, close to values observed in real data in Landrace and Yorkshire breeds at the same distance (Boré *et al.* 2018).

Furthermore, the relationship between the two breeds (Cockerham 1969; Robertson 1975), defined as the covariance of allele frequencies was calculated as $8cov\left(p^{P1},p^{P2}\right)$ (Garcia-Baccino *et al.* 2017), where $p^{P1}$ and $p^{P2}$ are the allele frequencies across all loci of P1 and P2, respectively. The average of the relationship over replicates was equal to 0.24, close to that estimated between Landrace and Yorkshire base populations (0.26) (Xiang *et al.* 2017). Hence, the simulated breeds (recent populations) mimicked the structure of the real ones both within and between breeds.

### Simulation of heterosis and QTL effects

QTL positions were the same in P1, P2 and CB, but with different allele frequencies. The positions and effects of 2500 QTL were sampled anew in each replicate. A maternal trait (*e.g.*, litter size) controlled by additive and dominance QTL action was simulated. The assumed genetic model is the same as Xiang *et al.* (2016), which involves additive and dominance effects correlated among PB and CB populations, and genomic heterosis.

To simulate heterosis (superiority of heterozygotes over homozygotes), a possible model is directional dominance, which states that dominance effects tend (on average across all QTL) to be favorable for the trait, *i.e.*, $E\left(d\right)=\mu_{d}$. For ease of simulation, we split the dominance effect at each locus as $d=\mu_{d}+d^{*}$ such that $\mu_{d}$ is constant across all QTL loci and $d^{*}$ is a random deviation normally distributed with zero mean. From Xiang *et al.* (2016) it is known that the value of $\mu_{d}$ is obtained from estimates of heterosis (or of inbreeding depression) as $\mu_{d}=-\frac{b}{m}$, where b is the inbreeding depression parameter (or the value of heterosis if the sign is switched), and in our simulation m is the number of QTL. Note that there is still individual heterosis as for each individual the heterosis is equal to $\left(1-f_{g}\right)m\mu_{d}$, where $\left(1-f_{g}\right)$ is individual heterozygosity, and $f_{g}$ is the vector of genomic inbreeding coefficients, calculated as the proportion of homozygous QTL per individual. Here, we assumed that inbreeding depression parameters were equal to -10 (equivalent to -1 (*e.g.*, piglet) per 10% increase in genomic inbreeding) in P1, P2 and CB $\left(b^{P1},b^{P2},\;b^{c}\right)$, and were distributed across all QTL. As reference, estimates of inbreeding depression b reported in the literature for litter size ranged from -2 to -9 piglets per 100% of observed homozygosity (Xiang *et al.* 2016; Iversen *et al.* 2019).

Then we simulated breed-specific QTL effects, but correlated across the three populations (P1, P2 and CB) to account for differences due to GxE and gen-by-gen (GxG) (epistasis) interactions. To do that, additive $\left(\sigma_{u}^{2}\right)$ and dominance $\left(\sigma_{v}^{2}\right)$ estimated genetic variances from Xiang *et al.* (2016) for total number of piglets born were used. The values were equal to 0.86, 0.54 and 0.28 for $\sigma_{u}^{2}$; and 0.04, 0.06 and 0.02 for $\sigma_{v}^{2}$ in Landrace, Yorkshire and their crossbreds, respectively. Then, for each of the QTL locus, two 3 by 3 QTL covariance matrices (one for additive a and one for dominance $d^{*}$) were built using the estimated variances of Xiang *et al.* (2016) from above and assuming a correlation between QTL effects ($r_{QTL}$) of 0.5 across populations as described in the Supplemental Material, S1. The QTL additive and dominance effects ($a_{QTL}^{P1}$, $a_{QTL}^{P2}$, $a_{QTL}^{c}$ and $d_{QTL}^{*P1}$, $d_{QTL}^{*P2}$, $d_{QTL}^{*c}$) were sampled from two multivariate normal distributions with zero mean and covariance matrices described in the Supplemental Material, S1. The “overall” value of the QTL dominance effect was obtained summing back the random deviate to its mean as $d=\mu_{d}+d^{*}$.

Residual variances ($\sigma_{e^{P1}}^{2},\sigma_{e^{P2}}^{2},\sigma_{e^{c}}^{2}$) were assumed uncorrelated across populations and were adjusted to reach a heritability in narrow-sense ($h^{2}$) and the resulting proportions of dominance variance to phenotypic variance ($h_{d}^{2}$). Three cases with different values for $h^{2}$ and $h_{d}^{2}$ were simulated: (1) $h^{2}=0.1$ and $h_{d}^{2}=0.01$ (as in Xiang *et al.* (2016)), (2) $h^{2}=0.1$ and $h_{d}^{2}=0.1$ (large variation due to dominance), and (3) $h^{2}=0.3$ and $h_{d}^{2}=0.1$ (more heritable trait).

### Simulation of true genetic values and phenotype

Each individual in the two parental breeds (P1 and P2) has two true breeding values, one on the PB scale $\left(TBV_{P}\right)$ and one on the CB scale $\left(TBV_{C}\right)$. From the additive and dominance QTL effects, $TBV_{P}$ were computed according to Falconer (1981) for each individual i within each parental breed. For example for P1 ($TBV_{P_{i}}^{P1}$) was: $TBV_{P_{i}}^{P1}=\overset{nQTL}{\underset{j=1}{\sum }}(z_{ij}-2p_{QTL_{j}}^{P1_{f}})\alpha_{j}^{P1}$ where $\alpha_{j}^{P1}$ is the allele substitution effect for the $j^{th}$ QTL, in P1, calculated as $\alpha_{j}^{P1}=a_{QTL_{j}}^{P1}+d_{QTL_{j}}^{P1}\left(q_{QTL_{j}}^{P1}-p_{QTL_{j}}^{P1}\right)$ where $d_{QTL_{j}}^{P1}=d_{QTL_{j}}^{*P1}+\mu_{d}^{P1}$ is the dominance QTL effect from P1 breed including directional dominance $\mu_{d}^{P1}$ in addition to the random deviate $d_{QTL_{j}}^{*P1}$; and $z_{ij}$ is equal to 2, 1 or 0 when the QTL genotype for animal i is AA, Aa or aa, respectively. The allele frequency $p_{QTL_{j}}^{P1_{f}}$ for A was obtained from the founder population in P1, and the allele frequencies $p_{QTL_{j}}^{P1}$ and $q_{QTL_{j}}^{P1}$ for A and a, respectively, were computed at each generation.

The $TBV_{C}$ for one breed (*e.g.*, P1 $TBV_{C_{i}}^{P1}$) depends on the allele frequencies of the other breed (P2 with allele frequencies $p_{QTL_{j}}^{P2},\;q_{QTL_{j}}^{P2}$). For the pure breed P1, the substitution effect is $\alpha_{C_{j}}^{P1}=a_{QTL_{j}}^{c}+\left(q_{QTL_{j}}^{P2}-p_{QTL_{j}}^{P2}\right)d_{QTL_{j}}^{c}$, whereas for P2 this is $\alpha_{C_{j}}^{P2}=a_{QTL_{j}}^{c}+\left(q_{QTL_{j}}^{P1}-p_{QTL_{j}}^{P1}\right)d_{QTL_{j}}^{c}$ where $\alpha_{C_{j}}^{P1}$ is the additive effect of the gametes from P1 when crossed with P2, and $\alpha_{C_{j}}^{P2}$ is the additive effect of gametes from P2 when crossed with P1 (Vitezica *et al.* 2016). Note that here, additive $\left(a_{QTL_{j}}^{c}\right)$ and dominance $\left(d_{QTL_{j}}^{c}\right)$ QTL effects are from the CB population. Then, the breeding value on the CB scale for each individual i in P1 was computed as $TBV_{C_{i}}^{P1}=\overset{nQTL}{\underset{j=1}{\sum }}(z_{ij}-2p_{QTL_{j}}^{P1})\alpha_{C_{j}}^{P1}$, with a similar expression for P2 but using $p_{QTL_{j}}^{P2}$ and $\alpha_{C_{j}}^{P2}$ instead.

The true total genetic value (TTGV) was computed for CB animals. For instance, for an individual i in CB its TTGV was computed as:

$$TTGV_{i}=\overset{nQTL}{\underset{j=1}{\sum }}z_{ij}a_{QTL_{j}}^{c}+\overset{nQTL}{\underset{j=1}{\sum }}z_{ij}(2-z_{ij})d_{QTL_{j}}^{c}\;\;\;$$

Note that $z_{ij}(2-z_{ij})$ in the second term of the equation is equal to 0 or 1 for homozygous and heterozygous genotypes, respectively. The phenotype of the trait was calculated by adding a general mean equal to 10 and a random error to the total genetic value of each CB animal. Only females had records (like for litter size).

### Scenarios and prediction models

Four scenarios (S1, S2, S3 and S4) of selection were simulated. They differed in the sources of information used for genomic evaluation, in selection criteria within-breed and in mate allocation strategies used to create the CB descendants. Table 1 shows a short description of these scenarios. Scenarios S1 and S2 can be considered “classical” schemes since only purebred information was used to evaluate the selection candidates within each breed (P1 and P2). The evaluation model was an univariate GBLUP including additive genetic effects and genomic inbreeding. Only PB information (genotypes and phenotypes) was considered, and evaluations were performed within each parental breed. The selection criterion of PB animals was the genomic estimated breeding value on the PB scale ($EBV_{P}$). The CB populations were generated using either random mating (RM) (S1) or a mate allocation strategy (S2) that minimizes the average expected genomic inbreeding ($EFI_{ij}$). Scenarios S3 and S4 were “combined” schemes because they use PB and CB information (genotypes and phenotypes) and additive and dominance effects to perform the genetic evaluation. In these two scenarios, we used a multivariate model based on “biological” (genotypic) additive and dominance effects of SNPs and including genomic inbreeding. Both PB (P1, P2) and CB performances were treated as different but genetically correlated traits. The selection criterion of PB animals was the estimated genomic breeding value on the CB scale $\left(EBV_{C}\right)$. To create the CB population, S3 used RM, whereas S4 used a mate allocation strategy that maximized the average expected total genetic value ($ETGV_{ij}$) of the CB descendants. Genomic evaluation models for all scenarios are detailed in Supplemental Material, S2.

**Table 1:** Description of simulated scenarios

Scenario	Evaluation model	Source information	Selection criterion within breed	Creation of crossbred animals
S1	GBLUP	PB	$EBV_{P}$	RM
S2	GBLUP	PB	$EBV_{P}$	MA _min_$EFI_{ij}$
S3	Trivariate SNP-BLUP	PB and CB	$EBV_{C}$	RM
S4	Trivariate SNP-BLUP	PB and CB	$EBV_{C}$	MA _max_$ETGV_{ij}$

$EBV_{P}$ genomic estimated breeding value on the purebred scale.

$EBV_{C}$ genomic estimated breeding value on the crossbred scale.

PB purebred.

CB crossbred.

RM random mating.

MA _min_$EFI_{ij}$ mate allocation strategy that minimizes the average expected genomic inbreeding.

MA _max_$ETGV_{ij}$ mate allocation strategy that maximizes the average expected total genetic value.

As explained before, the 4 scenarios were tested across three cases of genetic parameters: (1) $h^{2}=$ 0.1 and $h_{d}^{2}=$0.01, (2) $h^{2}=0.1$ and $h_{d}^{2}=0.1$, and (3) $h^{2}=0.3$ and $h_{d}^{2}=0.1$. In all cases, the $r_{QTL}$ across loci for all pairs of populations (P1, P2 and CB) of functional additive and dominance effects was 0.5 which resulted in $r_{PC}$ (*i.e.*, $cor\left(TBV_{P},TBV_{C}\right)$) of 0.46, 0.30 and 0.42 (cases 1 to 3) in the founders generation. These $r_{PC}$ are the average of the two parental breeds, but values of $r_{PC}$ were very similar for P1 and P2.

Three extra scenarios were considered. Scenarios S1 (first) and S3 (second) were evaluated under case 3 considering a $r_{QTL}$ across loci of 0.8 (leading to $r_{PC}=0.68$). This was also explored to have a situation where there is little GxG or GxE interaction. The third one (S4*) considered S4, but using true QTL effects and genotypes instead of estimated SNP effects and genotypes, only to perform the mate allocation strategy. This gives the upper bound of the possibilities of mate allocation strategy.

### Mate allocation strategies

After selecting PB animals at each generation, two mate allocation strategies were tested in order to define the matings between males (from P1) and females (from P2) to create the CB animals. The first strategy (S2), aimed to minimize the average genomic inbreeding of the CB population. This strategy is commonly known as minimum coancestry mating. The second strategy (S4), was focused on maximizing the average total genetic value of the CB population by exploiting non-additive genetic effects. These two strategies were compared with the random mating used in S1 and S3.

For each of 2448 (12 males × 204 females) possible matings, the expected future inbreeding and the expected total genetic value of CB progeny were calculated. The expected future inbreeding ($EFI_{ij}$) of the progeny from a mating between the $i^{th}$ male (from P1) and the $j^{th}$ female (from P2), was calculated as the expected proportion of homozygous SNP loci across the genome, as follows:

$$EFI_{ij}=\underset{k}{\sum }\left[P_{ijk}\left(AA\right)+P_{ijk}\left(aa\right)\right]/N$$

where $P_{ijk}\left(AA\right)$ and $P_{ijk}\left(aa\right)$ are the probabilities of homozygous SNP genotypes AA, and aa, at the $k^{th}$ SNP; and N is the total number of SNP. Note that expected future heterozygosity is equal to $1-EFI_{ij}$, so minimizing genomic inbreeding is equivalent to maximizing heterozygosity.

The expected total genetic value $\left(ETGV_{ij}\right)$ of the CB progeny from the same mating was predicted according to Toro & Varona (2010), as follows:

$$ETGV_{ij}=\underset{k}{\sum }\left[P_{ijk}\left(AA\right){\hat{a}}_{k}+P_{ijk}\left(Aa\right){\hat{d}}_{k}+P_{ijk}\left(aa\right)(-{\hat{a}}_{k})\right]$$

where $P_{ijk}\left(Aa\right)$ is the probability of SNP genotype Aa from the progeny of mating ij at the $k^{th}$ SNP; ${\hat{a}}_{k}$ and ${\hat{d}}_{k}$ are the additive and dominance estimated effects of the $k^{th}$ SNP estimated in the CB. Again, ${\hat{d}}_{k}={\hat{d}}_{k}^{*}+\hat{\mu_{d}}$ includes inbreeding depression (or heterosis) in the form of $\mu_{d}$.

From all possible combinations of matings, we selected the best 204 matings that either minimized EFI (S2) or maximized ETGV (S4) of the CB descendants, depending on which mate allocation strategy was used. Optimization of matings was addressed via linear programming (Jansen and Wilton 1985) using the R (R-Core Team 2017) *lpsolve* package (Berkelaar *et al.* 2004). Two constraints were used in the optimization: (1) each male was mated to 17 females, and (2) each female could not be mated to more than one male. For instance, the linear programming function for $EFI_{ij}$ was defined as:

$$f_{min}\left(EFI_{ij}\right)=\overset{nm}{\underset{i=1}{\sum }}\overset{nf}{\underset{j=1}{\sum }}EFI_{ij}\times \theta_{ij},$$

where $\theta_{ij}$ are binary variables of decision, where 1 and 0 means that the mating between male i and female j is selected or not selected, respectively. Constrains for male i can be written as: $\theta_{i1}+\theta_{i2}+\theta_{i3}+\ldots +\theta_{i,nf}=17\;(i=1,2,\ldots ,nm)$, and for female j: $\theta_{1j}+\theta_{2j}+\theta_{3j}+\ldots +\theta_{nm,j}=1\;(j=1,2,\ldots ,nf)$, where nm and nf are the number of males (12) and females (204), respectively.

Scenarios were compared in terms of response to selection. The mean TTGV (or TBV) of CB (PB) animals was computed for each generation expressed relative to the mean TTGV (or TBV) at the generation 0 to evaluate the realized cumulative response to selection. Increased TTGV is primarily of interest, whereas TBV of PB animals is of secondary interest, meaning that although the most important objective in the crossbreeding scheme is boosting CB performance, PB lines need to be good enough to ensure its viability (*e.g.*, PB animals need a large litter size to produce enough dams for the next generations). Heterosis (H) was measured in the first and last generation for S1 and S3 in all cases of genetic variance component, as: $H=\overset{nQTL}{\underset{j=1}{\sum }}d_{QTL_{j}}^{c}{\left(p_{QTL_{j}}^{P1}-p_{QTL_{j}}^{P2}\right)}^{2}$ (Falconer 1981). The realized genomic inbreeding was also calculated for the three populations. Results were the average of the 10 replicates of each scenario.

### Data availability

Programs and simulated data are available at http://genoweb.toulouse.inra.fr/∼zvitezic/simuPB-CB_G3. A README file contains a description of the files, codes and programs; and general instructions to run the simulation. Supplemental material available at figshare: https://doi.org/10.25387/g3.12504638.

## Results

### Genetic correlation between PB and CB

The $r_{PC}$ values (*i.e.*, $cor\left(TBV_{P},TBV_{C}\right)$) in the founder and last generation of selection are presented in Table 2, for the four scenarios and all cases of genetic variance components. The $r_{PC}$ was the same in the founder generation for all scenarios within each case. In all cases and scenarios, $r_{PC}$ values in the last generation were lower than those in the founder generation.

**Table 2:** Genetic correlation $\left(r_{\mathrm{PC}}\right)$ between purebred and crossbred performances in the founder and last generation of selection for each scenario, under three cases of genetic variance component and $r_{\mathrm{QTL}}$= 0.5 and 0.8

				$r_{\mathrm{PC}}$ in founder generation*	$r_{\mathrm{PC}}$ in the last generation			
Case	$h^{2}$	$h_{d}^{2}$	$r_{\mathrm{QTL}}$		S1	S2	S3	S4
Case 1	0.1	0.01	0.5	0.46	0.36	0.36	0.32	0.34
Case 2	0.1	0.1	0.5	0.30	0.15	0.15	0.04	0.03
Case 3	0.3	0.1	0.5	0.42	0.28	0.28	0.18	0.22
Case 3	0.3	0.1	0.8	0.68	0.44		0.36	

* It is the same in all scenarios.

$h^{2}$ heritability in narrow-sense.

$h_{d}^{2}$ is the proportion of dominance variance to phenotypic variance.

$r_{QTL}$ is the correlation at the QTL level between purebred (P1, P2) and crossbred populations.

Scenarios: purebred selection is based on estimated breeding values on the purebred scale and the crossbreds are generated by either random mating (**S1**) or with mate allocation to minimizing the expected genomic inbreeding (**S2**); purebred selection is based on estimated breeding value on the crossbred scale accounting for dominance and the crossbreds are generated either by random mating (**S3**) or with mate allocation to maximize the average expected total genetic value of the progeny (**S4**).

As selection proceeds, the $r_{PC}$ value decreases over generations because the difference in allele frequencies between breeds increases (Wientjes and Calus 2017; Duenk *et al.* 2020). For instance, in case 2, the average absolute difference in allele frequencies between breeds in the founder generation was 0.21 in both S1 and S3, whereas in the last generation of selection it was 0.36 and 0.38 in S1 and S3, respectively. The most important reduction in $r_{PC}$ was observed in case 2 when PB animals were selected based on $EBV_{C}$ (from 0.30 to 0.04). This result agrees with Duenk *et al.* (2020) that showed that an increase in magnitude of dominance (as in case 2) results in a reduction in $r_{PC}$.

### Response to selection in crossbred performance

**Figure 4:**
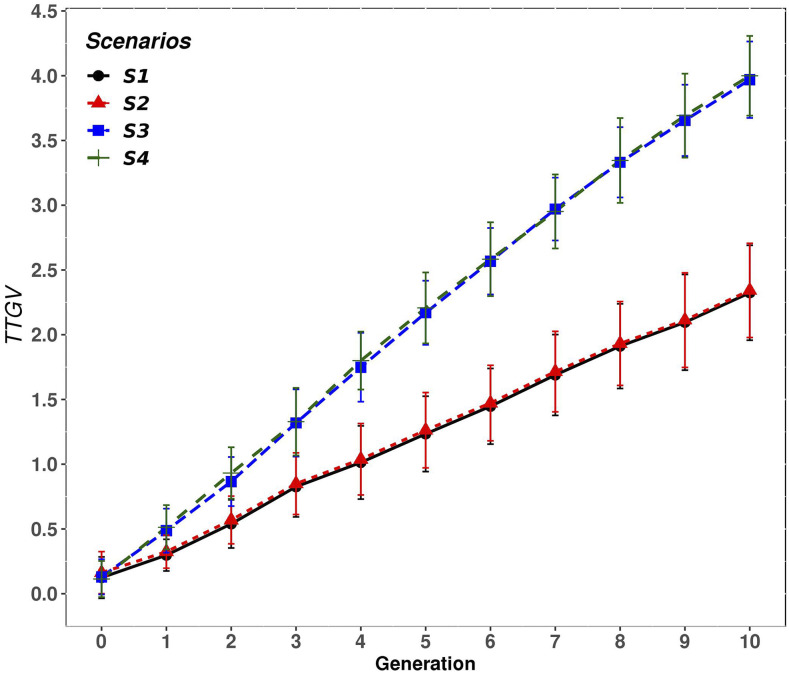
Accumulated response to selection (TTGV) of the crossbred animals for each generation and scenario expressed relative to generation 0, for case 1 ($h^{2}=$ 0.1 and $h_{d}^{2}=$ 0.01) and $r_{\mathrm{QTL}}=0.5$. Scenarios: purebred selection is based on estimated breeding values on the purebred scale and the crossbreds are generated by either random mating (S1) or with mate allocation to minimizing the expected genomic inbreeding (S2); purebred selection is based on estimated breeding value on the crossbred scale accounting for dominance and the crossbreds are generated either by random mating (S3) or with mate allocation to maximize the average expected total genetic value of the progeny (S4).

Figure 4 shows the mean TTGV of the CB animals accumulated across generations for the four scenarios (S1, S2, S3, S4), for case 1 $h^{2}=$ (0.1 and $h_{d}^{2}=$0.01) and $r_{QTL}=0.5$. Similar results were observed for the other cases of genetic variance components ($h^{2}=0.1$ and $h_{d}^{2}=0.1$, $h^{2}=0.3$ and $h_{d}^{2}=0.1$), they were not plotted here. Results (in Figure 4) show that scenarios S3 and S4, where PB and CB information was used and PB animals were selected on $EBV_{C}$, clearly outperform scenarios S1 and S2, where only PB information was used and the selection criteria was based on $EBV_{P}$, regardless of whether a mate allocation strategy was used or not. This advantage in S3 and S4 over S1 and S2 was observed from the first generations and the gap increased through generations.

The mean TTGV of CB animals accumulated in the last generation for each scenario in all cases of genetic variance components are presented in Table 3. The improvement in CB performance due to the selection criteria used $(EBV_{C}$ instead of $EBV_{P})$ was observed by contrasting S3 *vs.* S1. For $r_{QTL}=0.5$ the improvement was equal to 71%, 271% and 123%, for cases 1, 2 and 3, respectively (Table 3). An increase in heritability (*i.e.*, case 2 *vs.* case 3) resulted in a higher response to selection in CB performance in all scenarios, whereas the increase of dominance variance (*i.e.*, case 1 *vs.* case 2) was more advantageous for S3 and S4 than for S1 and S2. In fact, in S3 and S4, the proportion of improvement in CB performance was roughly tripled in case 2 (271%), where the ratio between dominance and additive variances was equal to 1 ($h^{2}=0.1$ and $h_{d}^{2}=0.1$) and $r_{PC}$ (0.30) was lower than the other cases. Thus, the benefit of using both PB and CB information and select PB animals based on $EBV_{C}$ (*i.e.*, S3 and S4) improved with the ratio dominance/additive variance. In scenarios where only PB information was used and the selection criteria was based on $EBV_{P}$ (*i.e.*, S1 and S2), some proportion of the additive genetic gain obtained on the PB breeds is transmitted to the CB progeny depending on the purebred-crossbred genetic correlation $r_{PC}$. For instance, in S1 and S2, where only PB information was used, a reduction in the mean TTGV of CB animals was observed when $h_{d}^{2}$ was increased from 0.01 to 0.1 and $h^{2}$ was held at 0.1 (*i.e.*, case 1 *vs.* case 2). This is because, under case 2, the increase in the proportion of dominance variance lowers the $r_{PC}$ from 0.46 to 0.30 in the founder generation (Table 2). This reduction in $r_{PC}$, in turn, reduces the proportion of genetic gain that is transmitted from PB parents to their crossbred descendants. However, when $h^{2}$ was increased from 0.1 to 0.3 and $h_{d}^{2}$ was held at 0.1 (*i.e.*, case 2 *vs.* case 3), and the $r_{PC}$ was 0.42, the mean TTGV of CB animals was higher compared to the case 1. These results show the importance of including CB information in the model to evaluate PB animals for CB performance, especially in cases where additive effects are low and dominance effects are relevant (like in case 2).

**Table 3:** Mean true total genetic value (standard deviation) of crossbred animals at the last generation for each scenario, under three cases of genetic variance component and $r_{\mathrm{QTL}}$= 0.5

Case	$h^{2}$	$h_{d}^{2}$	$r_{\mathrm{PC}}$	S1	S2	S3	S4
Case 1	0.1	0.01	0.46	2.32 (0.37)	2.34 (0.36)	3.97 (0.30)	4.00 (0.31)
Case 2	0.1	0.1	0.30	1.35 (0.40)	1.38 (0.41)	5.01 (0.40)	5.21 (0.44)
Case 3	0.3	0.1	0.42	2.48 (0.35)	2.50 (0.35)	5.54 (0.35)	5.57 (0.23)

$h^{2}$ heritability in narrow-sense.

$h_{d}^{2}$ is the proportion of dominance variance to phenotypic variance.

$r_{PC}$ is the average purebred-crossbred genetic correlation of the two parental breeds calculated in the founder generation.

Scenarios: purebred selection is based on estimated breeding values on the purebred scale and the crossbreds are generated by either random mating (**S1**) or with mate allocation to minimizing the expected genomic inbreeding (**S2**); purebred selection is based on estimated breeding value on the crossbred scale accounting for dominance and the crossbreds are generated either by random mating (**S3**) or with mate allocation to maximize the average expected total genetic value of the progeny (**S4**).

**Figure 5:**
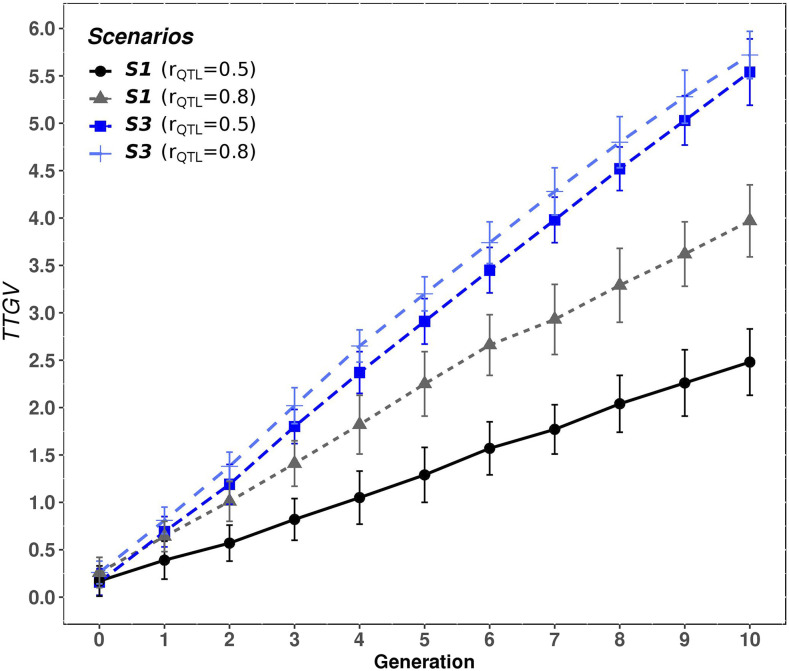
Accumulated response to selection in crossbred performance (TTGV) for scenarios S1 and S3 at different $r_{\mathrm{QTL}}$ (0.5 and 0.8) evaluated under case 3 ($h^{2}=$ 0.3 and $h_{d}^{2}=$ 0.1). In S1, purebred selection is based on estimated breeding values on the purebred scale and the crossbreds are generated by random mating. In S3, purebred selection is based on estimated breeding value on the crossbred scale accounting for dominance and the crossbreds are generated by random mating.

A correlation (at the QTL level) between PB and CB populations $\left(r_{QTL}\right)$ equal to 0.5 was assumed in the results mentioned earlier, which resulted in relatively low $r_{PC}$ correlations of 0.3 – 0.46. For case 3, a $r_{QTL}=0.8$, resulting in $r_{PC}=0.68$ was also examined. Figure 5 compares the effect of increasing the $r_{QTL}$ from 0.5 to 0.8 on the mean TTGV of CB animals for S1 and S3 evaluated under case 3. For $r_{QTL}=0.8$ ($r_{PC}=0.68)$ and case 3, the CB performance accumulated in the last generation for S1 and S3 were higher compared when $r_{QTL}=0.5$. The CB performances, accumulated in the last generations, were 2.48 and 3.97 in S1, and 5.54 and 5.72 in S3, for $r_{QTL}$ equal to 0.5 and 0.8, respectively. Thus, the increase in $r_{QTL}$ was reflected in an improvement in the CB performance in both scenarios, S1 and S3, but the improvement was greater in S1, where the selection was based on $EBV_{P}$. The advantage in CB performance of S3 over S1 was reduced from 123% at $r_{QTL}=0.5$ to 44% at $r_{QTL}=0.8$.

These results show, on one hand, that the proportion of the genetic progress that is transmitted from the parental breeds to their CB descendants, by selecting PB on $EBV_{P}$, depends on the value of $r_{PC}$, and on the second hand, the use of crossbred information to evaluate the PB for CB performance greatly helps the genetic improvement of the CB, especially if $r_{PC}$ is low.

### Change in genomic inbreeding and heterosis in purebreds and crossbreds

**Figure 6:**
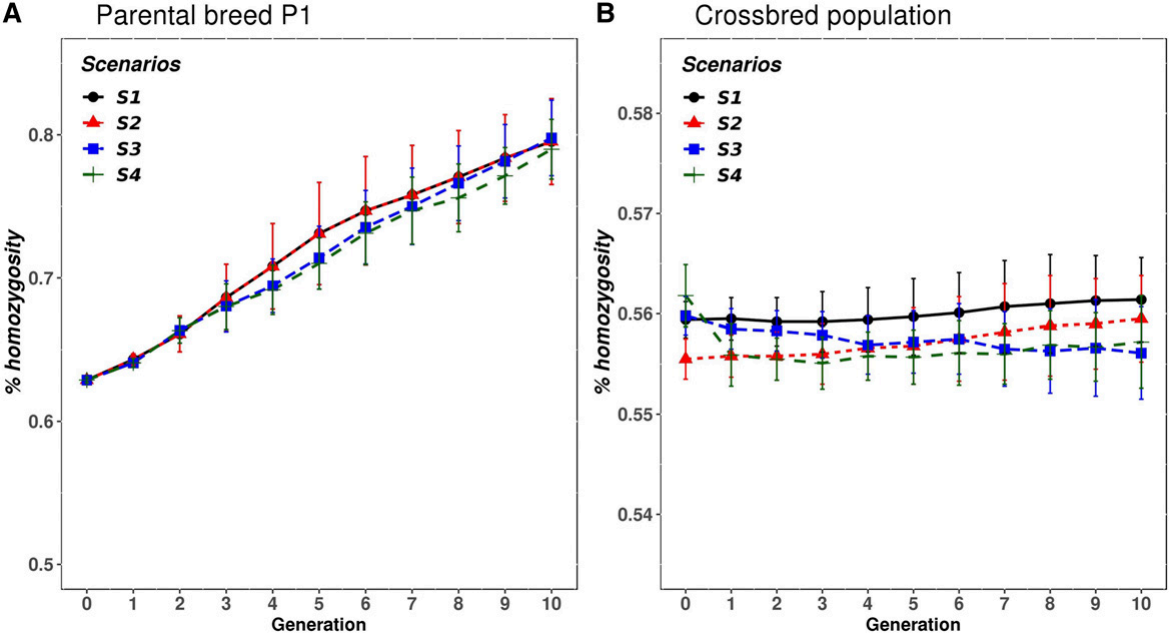
Mean genomic inbreeding for A) purebred animals from parental breed P1, and B) CB animals, for the four scenarios across each generation, for case 1 and $r_{\mathrm{QTL}}=0.5$. Scenarios: purebred selection is based on estimated breeding values on the purebred scale and the crossbreds are generated by either random mating (S1) or with mate allocation to minimizing the expected genomic inbreeding (S2); purebred selection is based on estimated breeding value on the crossbred scale accounting for dominance and the crossbreds are generated either by random mating (S3) or with mate allocation to maximize the average expected total genetic value of the progeny (S4).

Figure 6 shows the average, across animals, of genomic inbreeding (proportion of observed homozygosity) in P1 and in the CB population, for the four scenarios under case 1. Similar results were obtained for P2 and for the other cases (not shown). The genomic inbreeding of the two parental populations was around 0.62 in generation 0. The increase in genomic inbreeding per generation in the two parental breeds was almost the same in all scenarios, regardless of whether the selection criteria was in $EBV_{P}$ (S1 and S2) or $EBV_{C}$ (S3 and S4). For instance, the genomic inbreeding in P1 in the last generation was around 0.80 in all scenarios (Figure 6 A).

In the CB population, the initial average genomic inbreeding was around 0.56, which reflects a reduction of 0.06 in genomic inbreeding compared to the parental breeds (0.62 in PB – 0.56 in CB). Regardless of the scenarios and cases under investigation, genomic inbreeding remained almost the same across generations in the CB population. In scenarios where mate allocation strategies were implemented, a very small reduction in the genomic inbreeding was observed (Figure 6 B).

Estimates of inbreeding depression (or heterosis if the sign is changed) were close to the simulated value (-10 per 100% homozygosity) in the two parental breeds and lower in CB population. For instance, inbreeding depression was estimated in case 3 (standard deviation), as -13.3 (1.05) and -10.6 (1.0) for S1 and S3, respectively, in P1, and -5.94 (2.1) for CB in S3.

Heterosis was also computed at the first and last generations from QTL frequencies and the dominance effect. Both scenarios (S1 and S3) had the same initial amount of heterosis, 0.71, 0.75 and 0.73 for cases 1 to 3, respectively. The heterosis in the last generation for S1 was 2.24, 1.88 and 1.99, and for S3 was 2.49, 5.77, 3.66, for cases 1 to 3, respectively. The absolute value of the QTL frequency differences $\left|\left(p_{QTL_{j}}^{P1}-p_{QTL_{j}}^{P2}\right)\right|$ in the last generation were 0.367, 0.359 and 0.357 for S1 and 0.372, 0.379 and 0.373 for S3 in the last generation. Differences in heterosis are due to differences in QTL allele frequencies (between scenarios) and QTL dominance effects (among cases). Results showed that selecting PB on $EBV_{C}$ produced higher heterosis than selection on $EBV_{P}$, and it was higher when the ratio between dominance and additive variances was equal to 1 (*i.e.*, case 2).

### Effect of mate allocation strategies

Scenarios that differed in the use or not of mate allocation, but shared similar genomic evaluation model and selection criterion (*e.g.*, S2 *vs.* S1) were contrasted in order to measure the effect of using a mate allocation strategy. The advantage of using mate allocation strategies (S2 and S4) to improve CB performance was negligible compared to random mating (S1 and S3). On one hand, S2 based on minimizing the average expected genomic inbreeding of the CB progeny produced a small improvement on the CB performance (0.9, 2.2 and 0.8% for cases 1 to 3, respectively) compared to random mating (S1) (Table 3). This is probably because a very small reduction (0.5% in all cases) in the average genomic inbreeding (or increase in individual heterosis) of the CB population was observed in S2 compared to S1 (Figure 6). On the other hand, S4 based on maximizing the average expected total genetic value of the CB progeny, produced a slight increase in the CB performance (0.8, 4 and 0.5% for cases 1 to 3, respectively) compared to random mating (S3) (Table 3). The most important advantage of mate allocation was observed in S4 (4%) and in case 2, where the ratio dominance/additive variance was higher.

**Figure 7:**
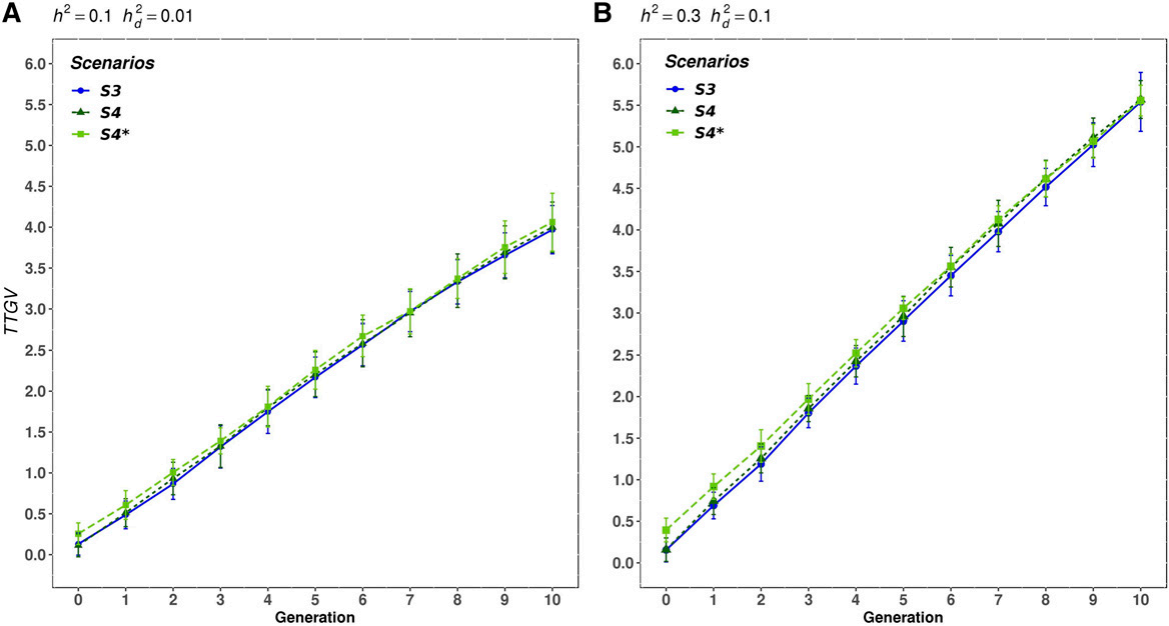
Mean true total genetic value of the CB animals for each generation for scenarios 3, 4, and the extra scenario (S4*), for A) case 1, B) case 3. Scenarios: purebred selection is based on estimated breeding value on the crossbred scale accounting for dominance and the crossbreds are generated by random mating (S3) or by mate allocation to maximize the average expected total genetic value of the progeny (S4). S4* uses the genotypes and effects of QTL, instead of the estimated SNP effects (as in S4), to implement the mate allocation strategy.

In order to get more insight on the mate allocation strategies, we created an extra scenario by replicating the S4 but instead of using the SNP genotypes and estimated SNP effects, we used the genotypes and effects of QTL to perform the mate allocation strategy (S4*). This situation represents the maximum achievable gain in CB performance by implementing this mate allocation strategy. Figure 7 shows the mean TTGV of CB animals accumulated across generations for S3, S4, and the extra scenario S4* where, to simplify, we only presented cases 1 and 3. The results show that assuming the QTL information as known (S4*), an increase of 0.13 and 0.24 in the CB performance was observed in the first generation in both cases, however, that improvement was gradually decreasing across generations of selection. Thus, in the last generation, the CB performance obtained in S4* (4.06, 5.29 and 5.56, for case 1, 2 and 3, respectively) was very similar to those obtained using the SNP effects and genotypes to perform mate allocation (S4, Table 3). Even when $h^{2}$ increased from 0.1 to 0.3 (case 3 *vs.* case 1) (Figure 7), the CB performance obtained in S4 was closer to that obtained in S4* (by using QTL effects), in the last generations. These results suggest that, even if the QTL genotypes and effects are known and used to perform mate allocation, the improvement in CB performance would be only in the first few generations of selection when the selection criteria is based on $EBV_{c}$.

### Response to selection in purebred animals

**Figure 8:**
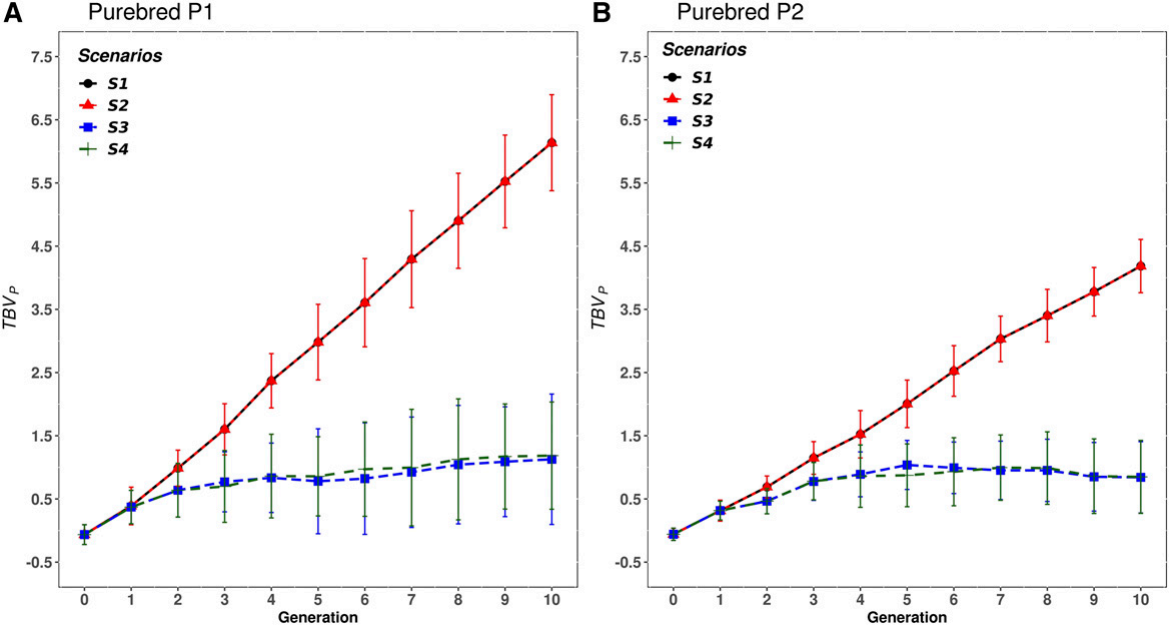
Mean true breeding value ($\mathrm{TB}V_{P}$) of the purebred animals from population A) P1 and B) P2, for each scenario and generation, for case 3 ($h^{2}=$ 0.3 and $h_{d}^{2}=$ 0.1) and a correlation at the QTL level of $r_{\mathrm{QTL}}=0.5$. Scenarios: purebred selection is based on estimated breeding values on the purebred scale and the crossbreds are generated by either random mating (S1) or with mate allocation to minimizing the expected genomic inbreeding (S2); purebred selection is based on estimated breeding value on the crossbred scale accounting for dominance and the crossbreds are generated either by random mating (S3) or with mate allocation to maximize the average expected total genetic value of the progeny (S4).

Figure 8 illustrates the accumulated response to selection ($TBV_{P})$ of PB animals from P1 and P2 across generations, for the four scenarios, case 3 ($h^{2}$=0.3 and $h_{d}^{2}$=0.1) and $r_{QTL}=0.5$. Since results were similar for the other cases of genetic variance components, they were not plotted. When PB animals were selected based on $EBV_{C}$, *i.e.*, S3 and S4, the accumulated response to selection was clearly lower compared to when they were selected based on $EBV_{P}$ (*i.e.*, S1 and S2). Compared to P1, the accumulated response in P2 was lower in all scenarios. In S1 and S2 this difference between P1 and P2 was due to differences in genetic variances (P2 has smaller genetic variances than P1), whereas in S3 and S4, where CB information was included, this difference was also because the scheme was not symmetric (P2 males had no crossbred daughters, see Figure 3).

The genetic response ($TBV_{P}$) for the two parental breeds accumulated in the last generation for the four scenarios under the three cases of genetic variance components are presented in Table 4. Response to selection in PB was the same between S1 and S2, and also changed slightly between S3 and S4 (in case 1) due to the different mate allocation strategies used to produce crossbreds. No genetic response in S3 and S4 was observed in PB when the proportion of dominance variance $h_{d}^{2}$ increased (*i.e.*, case 1 *vs.* case 2). This absence of genetic response can be explained by a $r_{PC}$ equal to 0.04 and 0.03 in the last generation for S3 and S4 respectively (see Table 2). An increase in $h^{2}$ (*i.e.*, case 2 *vs.* case 3) resulted in a higher genetic response in P1 and P2 breeds in all scenarios.

**Table 4:** Mean true breeding value (standard deviation) of purebred animals in the purebred scale ($\mathrm{TB}V_{P}$) for the two parental breeds, at the last generation and for each scenario and all cases of genetic variance components and $r_{\mathrm{QTL}}$ = 0.5

Purebred	Case	$h^{2}$	$h_{d}^{2}$	$r_{\mathrm{PC}}$	S1	S2	S3	S4
P1	Case 1	0.1	0.01	0.46	4.30 (0.82)	4.30 (0.82)	1.06 (0.48)	1.35 (0.58)
	Case 2	0.1	0.1	0.30	3.25 (0.82)	3.25 (0.82)	−2.17 (1.37)	−2.30 (1.09)
	Case 3	0.3	0.1	0.42	6.14 (0.76)	6.14 (0.76)	1.13 (1.03)	1.19 (0.85)
P2	Case 1	0.1	0.01	0.46	3.10 (0.45)	3.10 (0.45)	1.32 (0.60)	0.93 (0.62)
	Case 2	0.1	0.1	0.30	1.42 (0.74)	1.42 (0.74)	−1.99 (0.57)	−2.04 (0.87)
	Case 3	0.3	0.1	0.42	4.19 (0.42)	4.19 (0.42)	0.84 (0.56)	0.85 (0.58)

$h^{2}$ heritability in narrow-sense.

$h_{d}^{2}$ proportion of dominance variance to phenotypic variance.

$r_{\mathrm{PC}}$ is the average purebred-crossbred genetic correlation of the two parental breeds calculated in the founder generation.

Scenarios: purebred selection is based on estimated breeding values on the purebred scale and the crossbreds are generated by either random mating (**S1**) or with mate allocation to minimizing the expected genomic inbreeding (**S2**); purebred selection is based on estimated breeding value on the crossbred scale accounting for dominance and the crossbreds are generated either by random mating (**S3**) or with mate allocation to maximize the average expected total genetic value of the progeny (**S4**).

**Figure 9:**
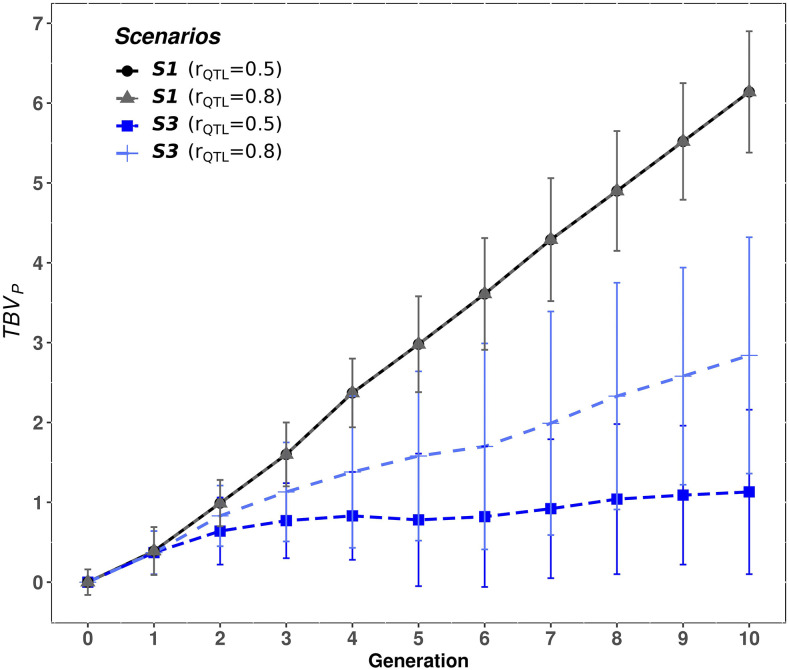
Accumulated response to selection ($\mathrm{TB}V_{P}$) in purebred P1 for scenarios S1 and S3, at different values of correlation at QTL level ($r_{\mathrm{QTL}}$ equal to 0.5 and 0.8) evaluated under case 3 ($h^{2}=$ 0.3 and $h_{d}^{2}=$ 0.1). In S1, purebred selection is based on estimated breeding values on the purebred scale and the crossbreds are generated by random mating. In S3, purebred selection is based on estimated breeding value on the crossbred scale accounting for dominance and the crossbreds are generated by random mating.

Additionally, Figure 9 shows the accumulated genetic response in P1 for two values of $r_{QTL}$ (0.5, 0.8) in S1 and S3 under the case 3 ($h^{2}$=0.3 and $h_{d}^{2}$=0.1). The accumulated genetic response in both parental breeds in S1 did not change with $r_{QTL}$, whereas an important increase was observed in S3 (from 1.13 to 2.84 in P1 and from 0.84 to 2.41 in P2) when $r_{QTL}$ was increased. Thus, the loss in $TBV_{P}$ by selecting PB animals on $EBV_{C}$ was reduced by almost half (*e.g.*, from 80 to 42% in P2) when $r_{QTL}$ increased from 0.5 to 0.8. This is as expected – selection for CB performance in S3 and S4 results in a correlated response in PB performance, and the magnitude of the response in PB performance depends on the value of $r_{PC}$.

## Discussion

The purpose of this work was to investigate, in a crossbreeding scheme, genomic evaluation models and the potential benefit of applying mate allocation. Novelties of our work from previous works include: (1) we simulate QTL effects that are correlated, but not identical, across populations, (2) we optimize matings in the crossbreds and (3) we consider in the simulation genetic evaluation using a multiple-trait SNP-BLUP across the three populations (purebreds and crossbreds) including additive and dominance effects.

To date, breed-specific QTL effects correlated across PB and CB have not been accounted for in simulations. Our results show, according to the literature, that a genomic evaluation model that explicitly includes dominance and phenotype and genotype data of PB and CB animals improves CB performance faster than a model considering PB data only, in particular for low values of $r_{pc}$. Finally, using mate allocation provides a negligible extra response in CB performance, even for cases where dominance variance was high.

All selection steps were within parental PB and mate allocation was only implemented to create CB population. Four scenarios that combined different sources of information (PB or PB and CB), selection criteria and mate allocation strategies were evaluated (over 10 generations) under different values of genetic variance components.

### Comparison of selection criteria

Our results show that selecting PB animals based on the $EBV_{C}$ produced higher CB performance compared to selection on the $EBV_{P}$, but at the cost of reducing the genetic response in the parental breeds. Our results agree with previous studies reported in the literature (Dekkers 2007; Esfandyari *et al.* 2015a; Wientjes and Calus 2017). Thus, choosing the selection criteria depends on the breeding objectives and the $r_{PC}$ correlation. If the PB trait is of interest, it is possible to weight both traits (PB and CB performance) in a selection index (*e.g.*, Esfandyari *et al.* (2018)).

The selection criteria (selecting on $EBV_{P}$ or on $EBV_{C}$) differ in how the allele substitution effects are calculated. For calculating the $EBV_{C}$ of PB, the allele substitution effects were computed using the SNP effects estimated for the CB population from the trivariate model. Thus, the effects of PB alleles are estimated on the genetic background and environment where they will be expressed in (Dekkers 2007; Ibánẽz-Escriche *et al.* 2009). Moreover, as the trivariate SNP-BLUP model provides estimates for both additive and dominance effects, it allows calibrating the substitution effect of one parent breed based on the allele frequencies of the opposite breed, with which it will be crossed, and conversely (Falconer 1981; Zeng *et al.* 2013; Esfandyari *et al.* 2018). The resulting allele substitution effects are breed-specific and can be interpreted as additive genetic effects of the gamete from one breed when crossed with the opposite breed (Vitezica *et al.* 2016). Previous studies in the literature reported an extra response in CB performance when allele substitution effects were estimated using additive and dominance effects and calibrated with the allele frequencies of the opposite breed, regardless of whether the training was based on PB or CB data (Zeng *et al.* 2013; Esfandyari *et al.* 2015b 2018).

Furthermore, the conventional $EBV_{P}$ of PB were estimated from performances and genotypes of each parental breed, which are supposed to be under the influence of the nucleus environment where PB animals are raised. Furthermore, allele frequencies observed within the breed were used in the genetic evaluation. Thus, the resulting estimates of $EBV_{P}$ are relevant to improve the PB performances in the nucleus environment (Dekkers 2007). However, the genetic response obtained within PB populations is only partially transmitted to the CB progeny according to the PB-CB genetic correlation $r_{PC}$. Therefore, the $r_{PC}$ is a relevant parameter for breeders to decide if data at the CB commercial level should be collected.

Allele substitution effects are a function of allele frequencies and additive and dominance effects (Falconer 1981). If there are QTL that expresses overdominance in CB, the performance of CB animals will be maximized when opposite alleles are fixed in both parental breeds (Esfandyari *et al.* 2018). In this study, the proportions of QTL that exhibited overdominance in the CB population were 15, 38 and 25% in case 1 to 3, respectively. For these QTL, alternate alleles will tend to be fixed in parental populations when selecting PB on $EBV_{C}$, but at the same time, alleles that are unfavorable for PB performance will tend to be fixed alternatively in the PB lines. That could explains why selection on $EBV_{C}$ produced a loss in response to selection in PB. This phenomenon of allele fixation has been well examined in previous simulation studies in animal crossbreeding context (Zeng *et al.* 2013; Esfandyari *et al.* 2018), but also in hybrid breeding in crops, like maize (Technow *et al.* 2014).

### Comparison of genetic evaluations models

In our simulation, genetic evaluation integrated PB and CB phenotypes and genotypes by using a trivariate SNP-BLUP model with additive and dominance effects (*e.g.*, S3). This model explicitly distinguishes between PB and CB data by modeling additive and dominance marker effects correlated among populations, therefore, the resulting estimated SNP effects are breed-specific. As dominance is involved in heterosis, its inclusion in the model was expected to be more efficient than pure additive models when PB animals are selected for CB performance, as it was demonstrated by Zeng *et al.* (2013) and Xiang *et al.* (2016). Previous studies in the literature and in a genomic context, based on simulation or real data, have never investigated simultaneously the effect of accounting for PB and CB data and additive and dominance effects on genetic gains in the long term (Dekkers 2007; Esfandyari *et al.* 2015b, 2015a 2018; Lopes *et al.* 2017). All these authors concluded that training on CB increases CB performance compared to training on PB separately or in a combined way. Nevertheless, none of them used all sources of information in the genomic evaluation. To our knowledge, only Xiang *et al.* (2016) used both PB and CB information and estimated additive and non-additive genetic effects on real data. One reason for the lack of studies even with simulation is its complexity.

Furthermore, inbreeding was taken into account (as a covariate) in genetic evaluation models, to correctly estimate dominance variance and heterosis / inbreeding depression, but also because it produces an improvement in the prediction of breeding values (Xiang *et al.* 2016). Another advantage of the SNP-based model is that solutions are SNP effect estimates which can be used directly into the mate allocation algorithms. Furthermore, we assumed as known the parameters of correlation of SNP effects (identical to the simulated correlation at QTL), however, these can be estimated from data.

If the objective is to improve the performance of CB, the trivariate genetic evaluation model is the optimal approach to select PB based on $EBV_{c}$. One of the limits of this approach is that its implementation requires phenotypic and genotypic data collected at the commercial CB level, which is not commonly available due to logistics and costs. Genotyping cost is still high to have CB females genotyped in routine but the decreasing trend may open some opportunities. Alternatively, to avoid recording phenotypes and genotypes of CB in routine, marker effects can be estimated using phenotypes and genotypes from a random sample of CB. These estimated SNP effects can be used for a few generations of selection (Dekkers 2007), but at a cost of reducing the selection response in CB performance due to loss of LD between SNP and QTL (Toro and Varona 2010; Esfandyari *et al.* 2015a).

### Genomic inbreeding in purebreds and heterosis in crossbreds

A substantial increase in genomic inbreeding in PB was observed in all scenarios. The main causes of this can be attributed to the small number of selected males per generation (12) in our study, and because in absence of information (*e.g.*, maternal scheme context), EBVs tend to be shrunk toward family means, so that related individuals are selected. Note that we used random mating to produce next generations within each PB. However, minimum coancestry mating can be used to manage PB inbreeding.

The reduction in genomic inbreeding (or increase in heterozygosity) (0.06) in CB animals respect the parental populations (0.62 in PB – 0.56 in CB), shows the benefit of crossbreeding schemes for exploiting the phenomenon of heterosis, even if PB inbreeding increases over generations. However, the amount of heterosis across generations of selection on PB, can vary depending on the selection criterion. In the last generation, the amount of heterosis was higher when PB animals were selected on $EBV_{C}$ than on $EBV_{P}$. These results have also been observed in previous simulation studies (Zeng *et al.* 2013; Esfandyari *et al.* 2015a).

### Mate allocation strategies

The effectiveness of mate allocation strategies accounting for non-additive genetic effects to improve CB performance was evaluated. On the one hand, minimizing the expected future inbreeding of the progeny (*i.e.*, S2) does not seem a promising mate allocation strategy to boost CB performance. A slight reduction (0.5% for the three cases) in the CB genomic inbreeding was observed compared to random mating (S1) which resulted in an almost negligible improvement of CB performance across generations. During the optimization process of the mate allocation, it was observed that the distribution of the expected future inbreeding from all the potential matings between the selected parents, had a very small standard deviation (*e.g.*, 0.558 ± 0.005 for case 3 in generation 1). Due to this small variation in the inbreeding, the reduction in realized genomic inbreeding achieved by this mate allocation strategy was very small. On the other hand, maximizing the average expected total genetic value of the CB (S4) through mate allocation might promote SNP heterozygosity, especially in those regions where there are QTL with favorable dominance effects and, hence, increase CB performance. The CB performance in S4 was slightly better compared to random mating in S3, except in case 2 where the improvement was 4% in the last generation. These results show that the benefit of implement mate allocation is better when the ratio of dominance to additive variances is higher. That was also observed by Toro and Varona (2010).

Furthermore, when QTL information was assumed as known, the increase in CB performance was notable in the first generations, but then, the improvement was gradually decreasing until reaching a CB performance close to that of S4 in the last generation. Thus, the potential improvement of mate allocation decrease in the long term when PB animals are selected on $EBV_{C}$. One reason that could explain such gradual decrease in CB performance is because opposite alleles that increase the proportion of favorable heterozygotes in the CB are already fixed in both parental breeds when PB animals are selected on $EBV_{C}$. Thus, the number of loci with favorable heterozygotes that are potentially optimizable is reduced over the generations of selection on $EBV_{C}$. Toro and Varona (2010) also found that the advantage of mate allocation over random mating disappears in subsequent generations of selection, but in a PB population.

In our study, a larger genome with 18 chromosomes of 120 cM each and 2500 QTL was simulated compared to other studies in the literature (*e.g.*, Toro and Varona 2010; Esfandyari *et al.* 2015a). Our more realistic genetic architecture led to very small effects of QTL, especially when genetic variances were small (as in case 1), in such a way that a large training set is required to estimate genetic effects (Zeng *et al.* 2013; Esfandyari *et al.* 2015a). In addition, our simulation included a maternal trait (only females had phenotype) and CB training population came from previous generations. Hence, in our simulation, it was more difficult to capture QTL effects through SNP than in previous studies, but it supposed to be closer to real situations. Our results agree with those obtained by Toro & Varona (2010) and Fernández *et al.* (2018), in the sense that the success of mate allocation strategies depends on the proportion of additive and dominance variance of the trait and the ability of estimating additive and dominance SNP effects.

The average expected increase in total genetic value of CB using mate allocation in S4, expressed in genetic (additive) standard deviations (SD), were 0.04, 0.21 and 0.13, for case 1, 2 and 3, respectively. Recently, we reported an increase in expected progeny performance of 0.09 SD, for the average piglet weight at birth within litter ($h^{2}=0.36$ and $h_{d}^{2}=0.04$) (González-Diéguez *et al.* 2019). Other studies in dairy cattle show increases from 0.8 to 0.13 SD and 0.1 to 0.22 SD for milk production and protein yield, respectively, with mate allocation (Ertl *et al.* 2014; Aliloo *et al.* 2017). None of these articles studied the benefits of implementing mating optimizations in the long-term in a crossbreeding scheme like ours. In our simulation, expected increases in CB performance were similar to those reported in the literature in single populations (PB).

In this study, first, PB animals were selected based on breeding values, and second, they were mated following different strategies to produce the CB animals. However, in this way, some matings with high progeny merit can be excluded (Hayes and Miller 2000). Another interesting strategy is mate selection (*i.e.*, deciding at the same time the selected individuals and their mates).

To conclude, our results show that a genomic evaluation model that simultaneously accounts for both PB and CB phenotype and genotype data and additive and dominance effects improves CB performance faster than a model considering PB data only, in particular for low values of $r_{pc}$. When $r_{PC}$ is low, selecting PB animals for CB performance using CB information is a more efficient strategy to exploit heterosis and increase performance at the CB commercial level. Furthermore, the benefit of mate allocation strategies for response in CB performance was negligible, even for cases where dominance variance was high. In addition, mate allocation implementation is not straightforward and will require some organizational changes (*e.g.*, semen logistic). The cost-benefit of implementing mate allocation is not clear.
